# The Development of Prosocial Attention Across Two Cultures

**DOI:** 10.3389/fpsyg.2019.00138

**Published:** 2019-02-05

**Authors:** Robert Hepach, Esther Herrmann

**Affiliations:** ^1^Department of Research Methods in Early Child Development, Leipzig University, Leipzig, Germany; ^2^Leipzig Research Center for Early Child Development, Leipzig University, Leipzig, Germany; ^3^Max Planck Institute for Evolutionary Anthropology, Leipzig, Germany; ^4^Minerva Research Group on the Origins of Human Self-Regulation, Max Planck Institute for Evolutionary Anthropology, Leipzig, Germany

**Keywords:** children, eye tracking, cross-cultural research, pupil dilation, attention

## Abstract

Despite the significance of prosocial attention for understanding variability in children’s prosociality little is known about its expression beyond infancy and outside the Western cultural context. In the current study we asked whether children’s sensitivity to others’ needs varies across ages and between a Western and Non-Western cultural group. We carried out a cross-cultural and cross-sectional eye tracking study in Kenya (*n* = 128) and Germany (*n* = 83) with children between the ages of 3 to 9 years old. Half the children were presented with videos depicting an instrumental helping situation in which one adult reached for an object while a second adult resolved or did not resolve the need. The second half of children watched perceptually controlled non-social control videos in which objects moved without any adults present. German children looked longer at the videos than Kenyan children who in turn looked longer at the non-social compared to the social videos. At the same time, children in both cultures and across all age groups anticipated the relevant solution to the instrumental problem in the social but not in the non-social control condition. We did not find systematic changes in children’s pupil dilation in response to seeing the problem occur or in response to the resolution of the situation. These findings suggest that children’s anticipation of how others’ needs are best resolved is a cross-cultural phenomenon that persists throughout childhood.

## Introduction

Prosocial attention, the degree to which we attend to the needs of others, precedes prosocial behavior. Even before they are old enough to actively help others themselves, children have been shown to focus on how well others are helped in both sharing and instrumental helping contexts (Kuhlmeier et al., 2003; Geraci and Surian, 2011; Hamlin et al., 2011; Hepach et al., 2016; Köster et al., 2016b). Seeing individuals being helped (or not) provides a child crucial social information. They become familiar with various forms of need, e.g., instrumental needs, emotional needs, and material needs (Dunfield, 2014), and they learn the prosocial or antisocial nature of the agents they are observing, i.e., whom to approach because they helped others and whom to avoid because they did not help others (Vaish et al., 2010; Dahl et al., 2013; Van de Vondervoort and Hamlin, 2018). Children’s prosocial *attention* is thus an important prerequisite for the maturation of their own prosocial behavior. Studying the mechanisms of prosocial attention, i.e., how children anticipate help and how their physiological arousal changes as a consequence of others needing help and being helped, can contribute to a better understanding of the individual differences observed in children’s prosocial behavior.

Infants are prosocially attentive from as young as 6 months. They expect resources to be distributed equally (Geraci and Surian, 2011; Schmidt and Sommerville, 2011; Sloane et al., 2012) and are surprised if others are blocked from achieving an instrumental goal and expect agents to approach those who hindered them over those who helped them (Kuhlmeier et al., 2003; Hamlin et al., 2007; Köster et al., 2016b). Infants not only form expectations about how others treat one another, but also prefer agents who have helped others over those who have harmed others (Hamlin et al., 2007). When making these choices, infants take into account an agent’s goal, avoiding those with harmful intentions even when they did not succeed in carrying out the harmful behavior (Hamlin, 2013). It has been suggested that sympathy in response to others’ distress underlies these social evaluations (Kanakogi et al., 2013). Infants not only respond to how others are helped but also anticipate how others are best helped (Köster et al., 2016b) and toddlers look longer at the correct solution to an agent’s instrumental problem (Hepach et al., 2016).

Prosocial behavior emerges in the second year of life across cultures (Callaghan et al., 2011). However, previous research shows that helping and sharing behaviors vary across culture and context (House et al., 2013; Blake et al., 2015; Paulus, 2015; Köster et al., 2016a). For instance, 1.5- to 2.5-year-old toddlers’ helping behavior varied in Germany and Brazil depending on how mothers structured helping tasks (Köster et al., 2016a). In a comparison of German and Indian children, the observed variability in instrumental helping was tied to parental scaffolding (Giner Torréns and Kärtner, 2017). With regards to sharing behavior, children varied in whether they engaged in costly sharing depending on their age and culture (House et al., 2013). Children’s prosociality undergoes major qualitative changes after 3 years of age with prosocial behavior changing in frequency and type. This raises important questions concerning the underlying mechanism. Does children’s attention to others’ needs increase or decrease with age or does it follow a u-shaped pattern similar to children’s sharing behavior (House et al., 2013; see also Blake et al., 2015)? Given the variability in children’s prosocial behavior across development and cultures, is there similar variability in prosocial attention (Kärtner, 2018)?

Children’s prosocial attention is closely tied to their prosocial behavior. Neural signature responses at 14 months were related to instrumental helping at 18 months and comforting at 14 months of age (Paulus et al., 2013). Twelve to 14-month-old infants’ expressed degree of the Nc ERP component in response to seeing others being helped or hindered related to whether or not they reached for the prosocial and antisocial agent (Cowell and Decety, 2015; see also Cowell et al., 2018). In addition to activation of the central nervous system, changes in the autonomous nervous system (ANS) activity predict whether and how much 1.5 to 5.5-year-old children will instrumentally help others (Hepach et al., 2017a) and how much they will share with others (Miller et al., 2015). More specifically, empathic concern but not personal distress predicts instrumental helping behavior in young children (Eisenberg, 2000). At 4 years of age, children’s baseline ANS activity and reactive ANS patterns predict their altruistic sharing (see Miller, 2018, for a recent review). As children enter school-age, sharing is related to behavioral control which becomes increasingly relevant for the self-regulation of selfish desires in order to benefit others (Steinbeis, 2018). Studying prosocial attention can thus provide important insights into of the mechanisms driving prosociality.

To date, the study of prosocial attention (as opposed to behavior) has focused on children at pre-weening age, typically younger than 3 years, and focused almost exclusively on Western samples. Therefore, this study extends previous work by e presenting German and Kenyan children aged 3 to 9 years old with a standardized eye tracking paradigm. The Kikuyu are the largest ethnic group in Kenya and E. H. has established a working relationship with local schools Kikuyu children are thus familiar with Westerners and not hesitant to participate in behavioral studies. The Kikuyu are traditionally small-scale farmers who cultivate vegetables and practice animal husbandry for their subsistence. The immediate nuclear family is the basic economic unit and relatives support one another. Many children attend the local nursery school from about 4 years of age, and almost all children in a community go to school once they are 5 years old.

Half the children were presented with videos that either depicted an instrumental helping situation in which one adult reached for an object while a second adult resolved the need or not. The other half watched perceptually controlled non-social videos in which objects moved without agents present. In the non-social videos each object followed the same movement trajectory as in the social condition. Following previous work by Hepach et al. (2016), we collected data in the social and non-social contexts based on four dependent variables: overall attention to the video stimuli, children’s looking time to the agent’s need prior to the resolution of the situation (see also Köster et al., 2016b), the change in children’s pupil dilation in response to seeing the need situation arise and the change in children’s pupil dilation upon seeing the situation being resolved. Such an assessment of children’s prosocial attention reduces the possible impact of children’s shyness in novel situations.

Pupillometry is an established measure of internal arousal in infancy and early childhood research (Laeng et al., 2012; Sirois and Brisson, 2014; Hepach and Westermann, 2016) similar to research in adults that shows greater pupil dilation in response to emotionally arousing images and sounds (e.g., Partala and Surakka, 2003; Bradley et al., 2008; Snowden et al., 2016). In the context of viewing others needing help, children’s increase in pupil dilation relates to whether and how fast they are to subsequently help (Hepach et al., 2016, 2017a). Assessing children’s pupil dilation *in response* to seeing others needing and being helped complements measures of children’s anticipatory looking behavior *before* others are helped. Taken together, these measures provide a window into children’s prosocial attention (Hepach et al., 2016; Köster et al., 2016b).

In the current study and based on prior work with 2-year-old German children, we predicted that children (1) look longer at the need in the social compared to the non-social condition (Hepach et al., 2016; Köster et al., 2016b), (2) show greater increase in pupil dilation in the social compared to the non-social condition (Hepach et al., 2016), and (3) that children’s internal arousal should decrease if the recipient’s need was fulfilled but remain elevated if the need was not appropriately fulfilled (Hepach et al., 2016). In addition, we explored whether children’s visual anticipation of the need resolution as well as children’s changes in pupil dilation varied with age. Second, we sought to apply pupillometry and anticipatory gaze tracking techniques within a cross-cultural research paradigm (German and Kenyan children) as for the most part these methods had only been used in studies with Western populations. We included cultural group as a fixed effect in each of the three analyses and did not have *a priori* predictions with regards to the direction of an effect of culture. Our statistical analyses of culture were thus exploratory.

## Materials and Methods

### Participants

**FIGURE 1:**
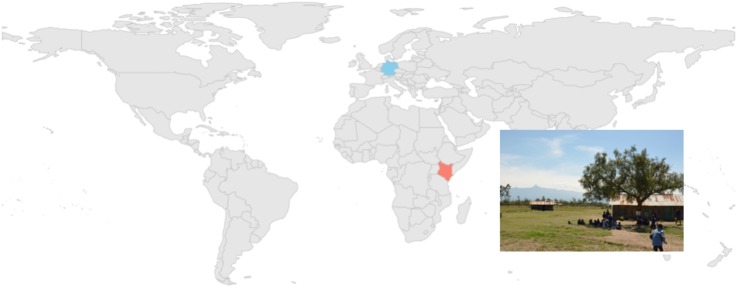
The two environments in which the eye tracking study was carried out in kindergartens in Leipzig, Germany and in the local schools around Nanyuki, Kenya. This figure was created using the R package rworldmap 1.3-6 (South, 2011).

Children were recruited in Leipzig, Germany and in local schools near Nanyuki in Kenya. German children came from middle-class families and Kenyan children were all Kikuyu, who lived in small villages near the Kenyan town of Nanyuki (see Figure 1). The German sample included 83 children (41 boys) and the Kenyan sample included 128 children (70 girls) across 7 age groups. Two additional German children were tested but excluded because one child was not tested in the correct experimental condition and because for one child the system sampled at a lower than average rate. Ten additional Kenyan children were tested but excluded because calibration could not be performed (*n* = 9) or because a child was not tested in the correct experimental condition.

This study’s design and procedure was carried out in accordance with ethical guidelines and ethical approval for this study was provided by the Max Planck Institute for Evolutionary Anthropology Child Subjects Committee. All parents were informed about the study and written consent to participate was obtained for each child from parents in Germany and from the children’s legal guardians (head teacher of the children’s schools) in Kenya. Both consent procedures were approved by the Max Planck Institute for Evolutionary Anthropology Child Subjects Committee that approved the study.

### Materials and Design

The videos were identical to those used in prior work (Hepach et al., 2016). In contrast to Hepach et al. (2016) we presented children with only one test trial and used only one type of neutral stimulus, i.e., the blue-colored set. We tested children in a full two factorial between-subjects research design. The independent factors were condition (social vs. non-social) and type of object returned (relevant vs. irrelevant object). Children were presented with videos of a (Western) adult male either stacking cans to build a tower or placing shoes onto a shelf. The adult was either observed by a (Western) adult female (social condition) or children watched videos of self-propelled items being stacked or placed without any adults present (non-social condition).

**FIGURE 2:**
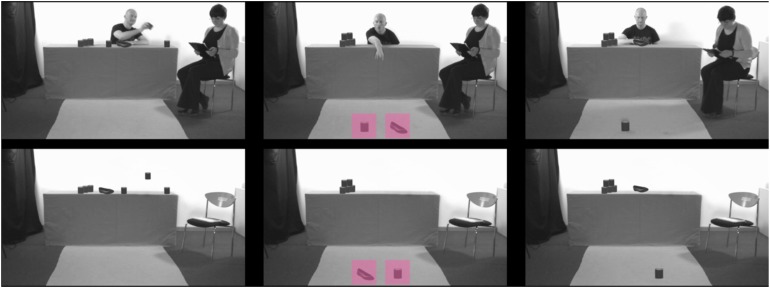
The key scenes of the social condition **(top)** and the non-social control condition **(bottom)**. The left panel depicts a frame from the introductory scene when the adult was stacking a tower (social condition) or a tower was being stacked (non-social condition). The center panel illustrates the key frame after the objects dropped to the floor. The regions of interest are marked here for illustration purposes. The right panel depicts the resolution of the situation after the second adult picked up an object (social condition) or after one object returned to the table. The individuals depicted here provided written consent for their images to be used in this figure.

Within each condition, the order of events was identical and proceeded as follows: first, in the *introductory video* (1120 gaze samples ∼ 19 s) the adult stacked the items (non-social condition: the items were being stacked). Second, in the *drop video* (1720 gaze samples ∼ 29 s) just before the task of stacking all items was complete, one relevant and one irrelevant object dropped to the floor. In the social condition only, the adult reached ambiguously for the items (no adults were featured in the non-social condition). In the final *resolution video* (750 samples ∼ 13 s) the second adult got up and handed the adult the irrelevant object (in the non-social condition the irrelevant object moved back on its own; see Figure 2). After each video, we presented the identical sequence of neutral stimuli on the computer screen throughout which pupil diameter was measured. These neutral videos were identical to those used in Hepach et al. (2016) and depicted computer animated bubbles on a blue background (see also Hepach et al., 2016, 2017a,b; Jessen et al., 2016). The total duration of the entire study for each participant was approximately 1 min and 40 s. Within each age group, we counterbalanced the type of context (social vs. non-social), the type of activity (stacking cans vs. placing shoes), and the position of the relevant object (left or right). Each child was presented with one video version. In sum, the trial children were presented with in the present study was identical to a trial used in Hepach et al. (2016) with the one exception that only the blue neutral measurement sequence was used.

During the study, children sat in front of an SMI eye tracking unit (model Red-m) attached below the screen of a laptop (17-inch; resolution 1,600 × 900 pixels). The sampling frequency of the eye tracker was set to 60 Hz. Stimuli were presented with Experiment Center (Version 3.7). The data of each child were exported from BeGaze (Version 3.7) to a text file. The processing and statistical analyses were carried out using *R* (Team, 2015).

### Procedure

In both Germany and Kenya children participated in the study in their respective schools. A female experimenter set up the laptop and eye tracker in a quiet room. She told children that she wanted to show them videos on a computer screen. Children were seated approximately 70 cm away from the laptop. For each child, we carried out a four-point standard calibration procedure. The experimenter remained seated next to the child during the experiment. Before children watched the actual study videos we presented a short video clip of a star image moving two four specified points on the screen in order to later recalibrate the position of children’s gaze (Frank et al., 2012). After children completed watching the videos they were escorted by the experimenter back to their respective play group.

## Data Analysis

We only included samples that belonged to a fixation, defined within BeGaze with 100px dispersion and 70 ms minimal duration. In addition, we averaged from the left and right eye for X and Y-data, respectively. For those 135 participants who provided data on the calibration videos the raw gaze data were additionally corrected using the procedure developed by Frank et al. (2012). The algorithm was adapted for *R* to *post hoc* correct participants’ point of gaze. For the remaining 75 participants (11 from the German sample) we included the data from the standard calibration of the eye tracking system.

Changes in children’s physiological arousal were assessed via changes in pupil dilation. The data were exported from the eye tracker and pre-processed in *R* (Version 3.4.1; Team, 2015) using the algorithms developed by Hepach et al. (2016). We measured children’s pupil dilation during each of the three presentations of the neutral video sequence. Specifically, each neutral video elicited two pupillary light reflexes in brief succession. We calculated the average pupillary minimum of the pupillary light reflex for each neutral video presentation. Increases in internal arousal results in an inhibited pupillary light reflex, therefore leaving the pupils more dilated (Steinhauer et al., 2000; Henderson et al., 2014; Hepach et al., 2015). In the present study, we calculated the change from baseline (first presentation of the neutral sequence) to after the drop sequence (process measure, second presentation of the neutral sequence) and the change from baseline to after the resolution scene (resolution measure, third presentation of the neutral sequence). The processing of pupil diameter changes and the identification of pupillary minimum was carried out in *R* and followed the steps reported in previous work (Hepach et al., 2012, 2016, 2017b).

The full data set including the text files exported from the eye tracking system, the processing scripts written in *R*, the data table which formed the basis of all the statistical analyses, and the *R*-script to execute those statistical analyses can be accessed at https://osf.io/wc3hr/.

### Looking Time: Initial Attention

To investigate children’s overall interest in the video before the objects dropped to the floor, we determined the time each child spent looking at the introductory sequence. More specifically, we calculated the number of samples that children looked at the respective video sequence (within the screen area of 1600 × 900 pixels) and divided this by duration of the sequence (1120 samples or 18.7 s) thus arriving at a proportion score for each participant. We ran an analysis of covariance (ANCOVA) including the interaction of condition (social vs. non-social) with the exploratory variable group (German vs. Kenyan) as well as the interaction of condition and age (linear and quadratic effect, z-standardized) while controlling for gender, and the type of game (can vs. shoes). Visual inspection indicated that model residuals were evenly distributed. This analysis included all 211 subjects who provided data on the introductory clip (see also Table 1).

**Table 1:** Participant overview.

Group	Condition	Object returned	Gender	Age group					
				
				3	4	5	6	7	8	9
**German sample** (n = 83/68/77/64)	**Social** (n = 42/30/37/28)	**Relevant** (n = 23/13/19/12)	**Girls** (n = 7/4/6/5)	1	0	2,0,1,1	1,1,1,0	1,0,1,1	0	2,2,2,2
			**Boys** (n = 16/9/13/7)	2,2,2,1	2,0,2,1	2,1,1,1	2,0,2,1	4,3,3,1	4,3,3,2	0
		**Irrelevant** (*n* = 19/17/18/16)	**Girls** (n = 14/12/13/12)	3	3	0	2,0,2,2	2	2,2,2,1	2,2,1,1
			**Boys** (n = 5/5/5/4)	0	1	1	1,1,1,1	0	0	2,2,2,1
	**Non-social** (n = 41/38/40/36)	**Relevant** (n = 17/14/16/15)	**Girls** (n = 9/7/8/8)	1	0	1	1	1,0,0,0	4,3,4,4	1,1,1,1
			**Boys** (n = 8/7/8/7)	1	2,1,2,2	2,2,2,1	0	1	0	2
		**Irrelevant** (n = 24/24/24/21)	**Girls** (n = 11/11/11/10)	1,1,1,0	3	2	2	2	0	1
			**Boys** (n = 13/13/13/11)	3	0	1	3	2	2,2,2,1	2,2,2,1
**Kenyan sample** (n = 128/76/77/59)	**Social** (n = 66/34/34/24)	**Relevant** (n = 38/17/17/13)	**Girls** (n = 16/7/7/5)	2,1,1,1	1	2	2,1,1,0	3,2,2,1	3,0,0,0	3,0,0,0
			**Boys** (n = 22/10/10/8)	4,3,3,1	2,0,0,0	2,1,1,1	2,2,0,0	4,1,2,2	6,2,3,3	2,1,1,1
		**Irrelevant** (n = 28/17/17/11)	**Girls** (n = 17/11/12/9)	3,2,1,1	3,3,1,1	1,1,1,0	2,0,2,2	3,2,3,2	3,1,2,2	2,2,2,1
			**Boys** (n = 11/6/5/2)	2,0,1,0	1,1,1,0	1	1,0,0,0	0	2,0,0,0	4,4,2,1
	**Non-social** (n = 62/42/43/35)	**Relevant** (*n* = 32/18/19/16)	**Girls** (n = 15/9/8/7)	1,1,0,0	1	1	1,0,1,1	4,0,0,0	4,3,3,2	3,3,2,2
			**Boys** (n = 17/9/11/9)	4,2,2,2	4,2,2,1	2,1,2,2	1,0,1,0	2,0,1,1	1	3,3,2,2
		**Irrelevant** (n = 30/24/24/19)	**Girls** (n = 12/11/10/9)	0	3,3,2,2	2	2	3,3,3,2	1,0,0,0	1
			**Boys** (n = 18/13/14/10)	3,3,2,0	2,1,1,0	1	3,2,3,3	2	4,1,3,2	3,3,2,2


*The values represent the number of participants used in the analysis of initial looking time/analysis of anticipatory looking/process pupil dilation/resolution pupil dilation. The analyses are thus based on different subsamples of participants. In case the same number of participants was used across all four analyses, a single number was entered in the respective cell.*

### Looking Time: Anticipatory Looking

We investigated children’s looking to the dropped objects within the crucial time window (13 to 29 s) in response to watching the *drop video*. For each child, we determined the time (i.e., found gaze samples) spent looking at each region of interest (ROI) encompassing the respective object. The dimensions and size of each ROI were identical and were adapted from the dimensions reported in Hepach et al. (2016) to the screen resolution of the present study (ROI width and height: 163 pixels). We calculated the dependent variable as the proportion of time children looked at the relevant object (time relevant object/[time irrelevant object + time relevant object]). Children were included in this analysis only if they looked either at the relevant or the relevant object ROI (see Figure 2 and Table 1). This analysis excluded children who looked at the screen but at neither ROI (see Table 1 for details on the number of children included in this analysis). We then ran an ANCOVA including the interaction of condition with the exploratory variable group as well as the interaction of condition and age (linear and quadratic effect, z-standardized) while controlling for gender, the type of game, as well as children’s initial visual attention (see analysis above). Plotting the distribution of residuals indicated a bi-modal distribution given that a majority of subjects either never or without exception looked at the relevant object after both objects had dropped. As a consequence, we carried out additional pair-wise comparisons using non-parametric Mann–Whitney-*U*-tests (with exact *p*-values). This analysis paralleled that of Hepach et al. (2016) and provided a test of our first research hypothesis (see Table 1 for details on the number of participants included in the analysis). To compare our results more closely to those reported by Hepach et al. (2016) we carried out focal analyses comparing children’s looking time to the relevant object between the social and non-social condition for German sample only.

### Pupil Dilation: Process Analysis

The change in children’s pupil dilation as a consequence of seeing the objects drop was assessed with an ANCOVA including the process change measure of pupil dilation as the dependent variable. The predictor variables were the interaction of condition and the exploratory variable group as well as the interaction of condition and age group (linear and quadratic function, z-standardized) while controlling for gender, and the type of game, children’s initial visual attention (see analysis above), as well as children’s baseline pupil diameter to account, indirectly, for different luminance levels across testing sessions (see Table 1 for details on the number of participants included in the analysis). The model residuals were normally distributed. This analysis paralleled that of Hepach et al. (2016) and provided a test of our second research hypothesis. Similar to our analysis of children’s anticipatory looking, we ran a focal analysis with the German sample to compare our results more directly to those obtained by Hepach et al. (2016).

### Pupil Dilation: Resolution Analysis

The change in children’s pupil dilation in response to the resolution of the situation was assessed with an ANCOVA including the change from process to the resolution measure of pupil dilation as the dependent variable. The predictor variables were the interaction of condition, type of object returned (relevant or irrelevant), and children’s process measure whilst controlling for the exploratory variable group, age (linear and quadratic function, z-standardized), gender, and type of game, and children’s initial attention (see Table 1 for details on the number of participants included in the analysis). The model residuals were normally distributed. This analysis paralleled that of Hepach et al. (2016) and provided a test of our third research hypothesis. To compare our results more closely to those reported by Hepach et al. (2016) we carried out a focal analysis for the German sample only.

## Results

### Looking Time

#### Initial Attention

**FIGURE 3:**
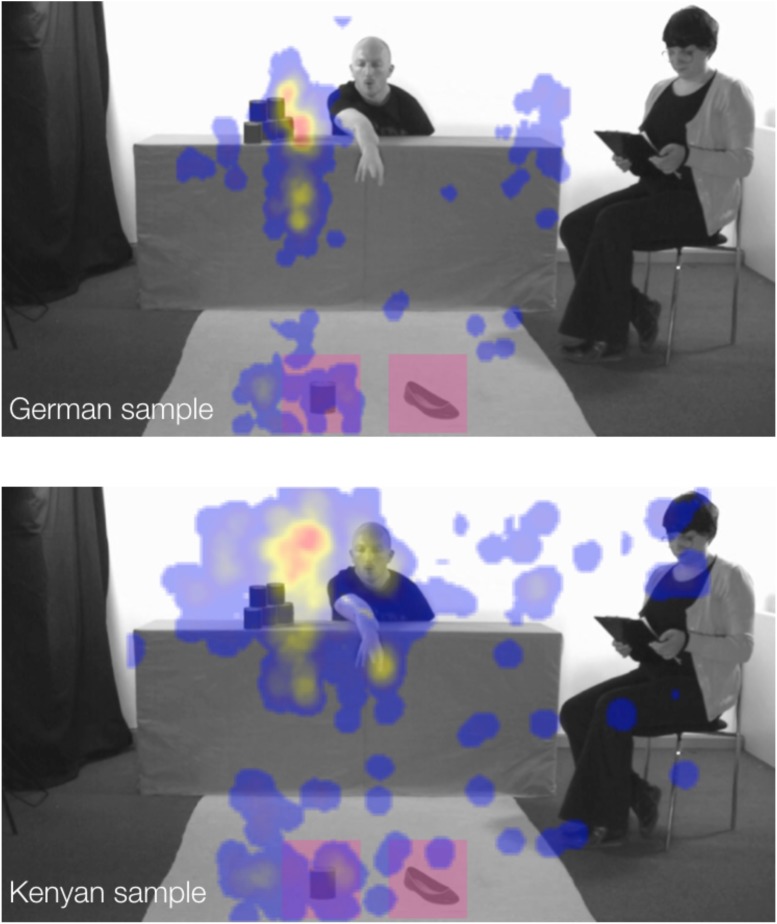
Visualization heat-maps to illustrate the distribution of attention across all age groups between the two cultural groups. Red color represents areas of greatest visual attention. The regions of interest are highlighted with red squares for the purpose of this illustration. The individuals depicted here provided written consent for their images to be used in this figure.

The time children spent looking at the video varied at a statistically marginal level by cultural group and condition, *F*(1,201) = 3.38, *p* = 0.069, $\eta_{p}^{2}$ = 0.02. Children in the German sample looked for similar lengths of time at the social (*M* = 0.75, *SD* = 0.26) compared to the non-social videos (*M* = 0.76, *SD* = 0.22), *t*(81) = -0.25, *p* = 0.81, 95% CI [-0.12, 0.09]. On the other hand, children in the Kenyan sample looked longer at the non-social (*M* = 0.57, *SD* = 0.28) than at the social videos (*M* = 0.42, *SD* = 0.3), *t*(126) = 2.93, *p* = 0.004, [0.05, 0.25] (see Figure 3). Overall, children from the German sample spent more time looking at the videos (*M* = 0.75, *SD* = 0.24) than children from the Kenyan sample (*M* = 0.49, *SD* = 0.3), *F*(1,201) = 46.37, *p* < 0.001, $\eta_{p}^{2}$ = 0.18, and children across groups looked longer at the non-social (*M* = 0.65, *SD* = 0.27) compared to the social videos (*M* = 0.55, *SD* = 0.32), *F*(1,201) = 6.72, *p* = 0.01, $\eta_{p}^{2}$ = 0.04. In addition and overall, children looked longer at the situation in which cans (*M* = 0.64, *SD* = 0.28) as opposed to shoes (*M* = 0.56, *SD* = 0.32) were stacked, *F*(1,201) = 4.3, *p* = 0.04, $\eta_{p}^{2}$ = 0.02. There were no interactions of age (linear or quadratic) and condition (*F*s < 0.7, *$\eta_{p}^{2}$ <* 0.01) and none of the remaining main effects reached statistical significance (*F*s < 3, $\eta_{p}^{2}$ < 0.01).

#### Anticipatory Looking

**FIGURE 4:**
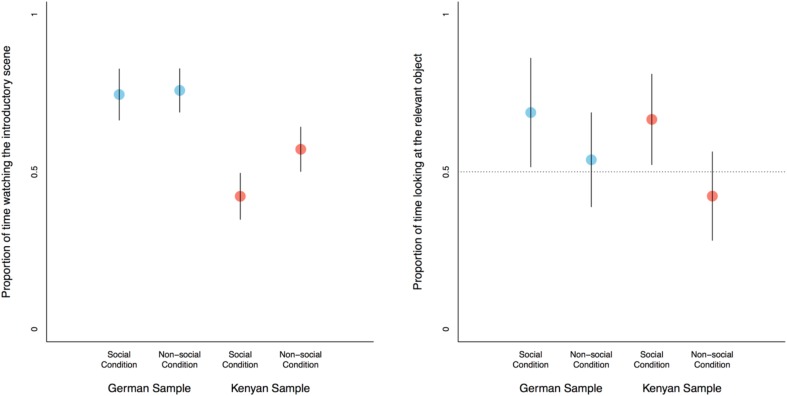
Overview of the results; means with 95% confidence intervals. **(Left)** The proportion of looking time with reference to the duration of the scene children spent on the initial introductory sequence before the objects dropped to the floor. German children looked longer at the videos than Kenyan children and Kenyan children looked longer at the non-social control videos than the social condition videos. **(Right)** The proportion of time children looked at the relevant object prior to the situation being resolved. Children in both cultural groups looked longer at the relevant object in the social compared to the non-social control condition.

The time children spent looking at the relevant object prior to the situation being resolved varied with condition, *F*(1,133) = 7.07, *p* = 0.009, $\eta_{p}^{2}$ = 0.05 (see Figure 4). Children looked longer at the relevant object in the social (*M* = 0.68, *SD* = 0.43) compared to the non-social condition (*M* = 0.48, *SD* = 0.45), *U(n*[social] = 64, *n*[non-social] = 80) = 3149, *p* = 0.01, 95% CI [0 0.31]. In addition, we found a marginally statistically significant main effect for game [*F*(1,133) = 3.44, *p* = 0.066, $\eta_{p}^{2}$ = 0.03] showing that children, across the social and non-social conditions, looked longer at the relevant object when shoes (*M* = 0.63, *SD* = 0.45) as opposed to cans (*M* = 0.5, *SD* = 0.45) were being stacked, *U(n*[can game] = 72, *n*[shoe game] = 72) = 3039, *p* = 0.056, 95% CI [-0.005 0.05]. None of the other main or interaction effects yielded statistically significant effects, *F*s < 2 and $\eta_{p}^{2}$ < 0.01. Our focal analyses of the German sample only yielded a no statistically significant difference between children’s anticipatory looking in the social (*M* = 0.69, *SD* = 0.46) compared to the non-social (*M* = 0.54, *SD* = 0.45) condition, *U(n*[social] = 30, *n*[non-social] = 38) = 688, *p* = 0.11, 95% CI [-0.02 0.3]. At the same time, German children looked at the relevant object more than 50% of the time only in the social condition, *T* = 321, *p* = 0.043, and not in the non-social condition, *T* = 400, *p* = 0.66.

### Pupil Dilation

#### Process Analysis

Children’s pupil dilation in response to seeing the objects drop did not vary as a function of cultural group, *F*(1,142) = 0.67, *p* = 0.42, $\eta_{p}^{2}$ < 0.01, or condition, *F*(1,142) = 2.77, *p* = 0.098, $\eta_{p}^{2}$ = 0.02. The analysis did yield a statistically significant main effect of children’s baseline pupil diameter, i.e., the larger children’s pupil during the baseline measurement sequence the smaller the change from baseline to after seeing the objects drop, β = -0.03, *SE* = 0.009, *t* = -3.04, *p* = 0.003. None of the interaction terms (*F*s < 2, $\eta_{p}^{2}$ < 0.01) or main effects had statistically significant effects (*F*s < 3, $\eta_{p}^{2}$ < 0.02). Our focal analyses for the German sample revealed that German children showed greater increase in pupil dilation in the social (*M* = 0.04, *SD* = 0.06) compared to the non-social (*M* = 0.02, *SD* = 0.06) condition, *F*(1,67) = 4.12, *p* = 0.046, $\eta_{p}^{2}$ = 0.06. In addition, we found that similar to our analyses of both samples, larger baseline pupil diameter was linked to smaller change in pupil dilation, β = -0.04, *SE* = 0.01, *t* = -3.5, *p* < 0.001. None of the interaction terms (*F*s < 1, $\eta_{p}^{2}$ < 0.01) or main effects (*F*s < 3, $\eta_{p}^{2}$ < 0.02) yielded statistically significant effects.

#### Resolution Analysis

Children’s pupil dilation in response to seeing the situation being resolved yielded a statistically significant effect of their process measure of pupil dilation. The greater children’s pupil dilation in response to seeing the objects drop, the smaller the change in pupil dilation after seeing one object return β = -0.67, *SE* = 0.15, *t* = -4.62, *p* < 0.001, a pattern that is consistent with values regressing to the mean. In addition, children’s pupil dilation remained increased in the situation showing cans compared to the situation showing shoes, *F*(1,109) = 3.97, *p* = 0.049, $\eta_{p}^{2}$ = 0.04. None of the remaining interaction terms (*F*s < 2, $\eta_{p}^{2}$ < 0.02) or main effects (*F*s < 3, $\eta_{p}^{2}$ < 0.03) yielded statistically significant effects. Our focal analyses for the German sample revealed a similar effect of children’s process measure of pupil dilation, β = -0.81, *SE* = 0.2, *t* = -3.97, *p* < 0.001. None of the interaction terms (*F*s < 2, $\eta_{p}^{2}$ < 0.03) or main effects (*F*s < 2, $\eta_{p}^{2}$ < 0.02) yielded statistically significant effects.

## Discussion

The current study is the first to compare children’s prosocial attention across 7 age groups from 3 to 9 years old and between a Western and Non-Western cultural group. The comparison of German and Kenyan children revealed that each group viewed the video stimuli differently. German children looked longer at the introductory sequence on the computer screen than Kenyan children. In addition, whereas German children attended equally to the social and non-social videos, Kenyan children spent more time looking at the non-social control videos than the social videos. These differences in initial overall attention may be explained by the difference in experience of watching computer animated clips without any human actors present. We found this difference in initial attention for the two cultural groups across the seven age groups. Crucially, despite different overall looking time to the introductory sequence, Kenyan and German children correctly anticipated the adult’s need. They fixated on the correct solution to the adult’s need more in the social compared to the non-social control condition across all age groups. This replicates previous work which focused predominately on Western children during the first 2 years of life (Hepach et al., 2016; Köster et al., 2016b). These findings suggest that children’s anticipation of how others’ needs are best resolved is a cross-cultural phenomenon that persists throughout childhood.

Previous work showed that changes in children’s internal arousal assessed via changes in pupil dilation, complemented findings from looking time analyses. Two-year-old children showed greater increase in physiological arousal when seeing others’ in need in a social compared to a non-social condition and pupil dilation remained elevated when the situation was not resolved appropriately (Hepach et al., 2016). In the present study, we did not find any systematic changes in children’s internal arousal across all participants but merely partial support for our second research hypothesis. Only German children showed a weak effect with more dilated pupils in response to seeing the objects drop in the social compared to the non-social control condition. These findings parallel ones with 2-year-old, German children (Hepach et al., 2016) but do suggest that the previously found effect does not generalize across age and cultural groups. Furthermore, we did not find support for our third research hypothesis. Children in the current in sample did not continue to show increased internal arousal when the adult was not helped thus failing to replicate a previous finding with 2-year-old German children (Hepach et al., 2016).

This deserves a detailed discussion given that we used the identical stimuli from Hepach et al. (2016). It is possible that the previously found effect of pupil dilation is specific to 2-year-old German children. The present study cannot rule out this possibility given that we did not have access to 2-year-old Kenyan children and thus did not test this age group. In fact, the central aim of the present study was to sample children 3 years of age and older and to apply the previously developed paradigm within a non-Western cultural group. The lack of statistically significant effects with regard to our assessment of children’s pupil dilation raises the question of whether the way in which we captured pupil diameter affected our results. The human pupil first and foremost responds to luminance changes, constricting to brighter stimuli and dilating within darker environments (Sirois and Brisson, 2014). We could not control the luminance levels during our experiment. In Kenya, the study room did not have electricity and the only light source was sun light through the windows (see Figure 1). Given that this was the first eye tracking experiment run at the study site we wanted children to be as comfortable as possible and decided to not alienate them from their familiar class room environment by darkening the room. Additionally, piloting in Germany showed that the specific SMI eye-tracker model we used in the study does not track the eyes well in dark rooms. Some light was thus needed in the study room which may have in turn interfered with the measurement. In Germany, we collected data in a comparable manner by not changing the luminance in the kindergarten room. The circumstances under which we collected pupil data were not ideal which may have impaired our ability to detect psychologically induced changes in pupil dilation.

At the same time, it is important to point out that the methodological constraint of not controlling luminance was not systematically confounded with age, group or condition because the measurement of pupil dilation was taken during the presentation of the neutral videos which were identical for all children. Thus, while we failed to control room luminance we did control screen luminance. Given that no prior work reported pupillometry findings in a cross-cultural study with human children, our study is the first to suggest that it is not enough to control for screen luminance during the measurement of pupil diameter and that control of room luminance is also required. We think that individual differences in room luminance across test sessions in our sample contributed to unaccounted for random measurement error thus failing to provide a strong test for rejecting the null-hypothesis of our second and third research hypotheses. We thus regard the lack of systematic difference in pupil dilation in the current sample to be methodological in nature, not psychological. Given the methodological concerns outlined above, we would caution against a strong theoretical conclusion on the basis of the pupillometry findings (or lack thereof). In addition, while this study represents the largest cross-cultural sample of children in a prosocial attention eye-tracking task, our final sample size within each age group was small. In comparison, previous work included 64 children for one age group of 2-year-old children (Hepach et al., 2016). We cannot rule out that a critical sample size is needed to detect systematic changes in pupil dilation. A necessary next step for future studies would be to conduct a cross-cultural comparison with more subjects per age group including 2-year-old children in a laboratory setting where both stimulus luminance and room luminance can be controlled.

In addition to assessing changes in children’s pupil dilation, we assessed their anticipatory looking behavior to test our first research hypothesis. The results of this analysis hold crucial theoretical value for the study of developing prosocial attention. We found that children across all seven age groups and both cultural groups looked longer at the solution that would correctly fulfill the adult’s needs. We can rule out that this was merely a perceptual preference given that no such anticipation was found in our non-social control condition. Across development, children continue to anticipate how others might best be helped. One avenue for future research is to assess both children’s prosocial attention as well as their prosocial behavior to understand the driving mechanism for these tendencies and to identify individual differences in children’s prosociality. Such an approach would also provide an opportunity to investigate different forms of prosocial behavior/attention. Whereas previous work focused on the variability of prosocial behavior across age groups and cultures in the context of sharing material resources (House et al., 2013; Blake et al., 2015), the study of children’s prosocial attention has focussed on instrumental need scenarios (Hepach et al., 2016; Köster et al., 2016b).

The current paradigm, with improved control of room luminance, lends itself well to cross-cultural investigations of prosocial attention. Disentangling socio-cognitive factors, i.e., children’s prosocial attention provides relevant information to better understand the emergence of prosociality (Callaghan and Corbit, 2018; Kärtner, 2018). Although instrumental helping behavior emerges early in ontogeny across cultures (Callaghan et al., 2011; Callaghan and Corbit, 2018) some other forms of prosociality such as sharing shows variability across development and cultures. An interesting focus for future research is the relation between prosocial attention and prosocial behavior. It is possible that a range of socio-cultural factors, such as maternal structuring of children’s instrumental helping behavior that has been found to differ between German, Brazilian, and Indian children, affect prosocial attention (Köster et al., 2016b; Giner Torréns and Kärtner, 2017; Kärtner, 2018). An alternative possibility, is that children’s sensitivity to others’ needs is less affected by socio-cultural factors than by prosocial behavior itself. This could suggest that culture affects not so much *whether* we perceive others’ needs but *how* we expect these needs to be fulfilled. In fact, cultures differ with respect to the norms that govern prosocial behavior but these norms may have a different impact on children’s prosocial attention (House et al., 2013). In one example, children’s aversion to unequal distributions in a resource allocation task followed different cultural and ontogenetic trajectories if the child (advantageous inequity aversion) or a peer (disadvantageous inequity aversion) benefited from the unequal distribution of resources (Blake et al., 2015). It is possible that children respond to both forms of inequity in all cultures, such as looking longer at the distribution or showing greater physiological arousal. But whether children intervene may depend more on developmental age and the norms of the culture they grow up in.

The current study is among the first to compare prosocial attention between a Western and non-Western culture as well as across multiple age groups. At the same time, there are a number of additional methodological improvements needed for future research. One explanation for why Kenyan children looked longer overall at the non-social stimuli than the social stimuli is because the social stimuli with Western adults were less interesting. It is possible that greater overall attention to the social stimuli would be achieved if Kenyan adults were depicted helping (or not helping) one another. Conversely, it would be interesting to present the German children with videos depicting Kenyan adults. For the purpose of the present study it is important to emphasize that we found both Kenyan and German children to look longer at the relevant object in the social than the non-social condition despite differences in their overall initial attention to the videos. Children in both cultures similarly anticipated how the adult would be best helped. Together our findings suggest children’s anticipation of others’ needs is a phenomenon that is not confined to the Western culture and persists throughout childhood. Future research will need to disseminate whether children’s prosocial attention and behavior follow different developmental trajectories.

## Author Contributions

RH and EH designed the study and wrote the paper. EH carried out data collection in Kenya and oversaw data collection in Germany. RH conducted the analyses.

## Conflict of Interest Statement

The authors declare that the research was conducted in the absence of any commercial or financial relationships that could be construed as a potential conflict of interest.
